# Improving team dynamics in an acute older people care unit to improve quality and safety of care

**DOI:** 10.1177/20503121251333314

**Published:** 2025-04-27

**Authors:** Rachid Akrour, Anja Pattschull, Suzana Stamenkovic, Catherine Courret Gilgen, Alberto Garcia Manjon, Yvan Bourgeois, Joachim Rapin

**Affiliations:** 1 Department of Internal Medicine, Service of Geriatrics Medicine and Geriatric Rehabilitation, Lausanne University Hospital and University of Lausanne, Switzerland; 2 Healthcare Direction, Lausanne University Hospital and University of Lausanne, Switzerland

**Keywords:** Patient care team, quality improvement, occupational health, work satisfaction, patient safety

## Abstract

**Introduction:**

Several studies have demonstrated that the team dynamic among healthcare professionals has an impact on the quality of care, as well as the satisfaction of healthcare team members at work. The Input-Process-Output and Intervention model is one way to structure complex interventions with the aim of improving the team dynamic and evaluating their impact on the healthcare professional team, patients, and the organization.

**Objective:**

The objective of the study was to describe the steps and results of a quality improvement project aimed at improving the team dynamic of healthcare professionals in the acute older people care unit of a university hospital and improving outcomes for the healthcare professional team, patients, and the organization.

**Method:**

The project was based on the Deming cycle framework (Plan-Do-Check-Act) for improving the quality of care. It consisted of the following steps: (1) Implementation plan with an iterative process; (2) interventions according to the Input-Process-Output and Intervention model; and (3) evaluation of the impact of these interventions.

**Results:**

The results demonstrated an improvement in most healthcare professional team outcomes, particularly in terms of satisfaction and absenteeism, as well as organizational outcomes. Patient outcomes were highly satisfactory and relatively stable throughout the process.

**Conclusion:**

The integration of a healthcare professional team dynamic improvement model into quality and safety of care processes should be considered to optimize clinical and organizational outcomes.

## Introduction

Evidence shows that the team dynamic in healthcare significantly influences the quality and safety of care and healthcare team satisfaction. A positive team dynamic can be correlated with decreased medical errors, falls, and infections, and increased patient and healthcare team satisfaction.^1,2^ Conversely, poor team dynamics leads to increased patient safety issues and substantial costs for healthcare systems.^1,3–6^ The quality and safety of care involve measuring the impact of care on clinical and organizational outcomes. Moreover, several studies have highlighted the importance of implementing nursing-sensitive indicators (NSIs) to improve quality and safety.^7,8^ Furthermore, implementing a system for monitoring care performance using nurse-sensitive indicators contributes to a more accurate reflection of the contribution of the healthcare team in terms of quality and safety, and to justify the resources necessary to maintain the constantly evolving level of care needed.^9^ NSIs can be categorized into four main groups: organizational-focused structural indicators, nursing-focused process/intervention indicators, nurse-focused outcome indicators, and patient-focused outcome indicators. Among the most frequently studied NSIs are mortality rates, failure to rescue, hospital-acquired infections (e.g., pneumonia, urinary tract infections, and wound infections), pressure ulcers, patient falls, and medication administration errors. Nurse-focused indicators such as job satisfaction, burnout, and turnover rates are also critical in understanding workforce stability and its impact on patient care.^8^

Song et al.^2^ defined an effective team dynamic according to three criteria: team performance, satisfaction of team members, and team adaptability. Moreover, the dynamics of healthcare teams should be considered within a broader sociopolitical context.^10^ Indeed, a complex intervention that establishes a healthy, collaborative, fair, flexible, and stimulating work environment with developmental opportunities enhances healthcare team engagement while reducing absenteeism.^11–13^ In addition, environment, culture, and organizational structure can be adapted and modified by team members to foster a dynamic and collaborative environment for shared values.^14^

The Input-Process-Output and Intervention (IPOI) model, developed by Körner et al.,^15^ proposes complex interventions in interprofessional teams to improve their dynamic. The authors suggest various measures to evaluate the impact of these interventions on healthcare teams, patients, and organizations. The authors identify six types of interventions to enhance and support the team dynamic: (1) complex interventions (combined intervention strategies); (2) implementation of tools (e.g., communication tools such as situation-background-assessment-recommendation or documentation); (3) reorganization of team composition (e.g., creation of new roles in the team); (4) restructuring team meetings; (5) training programs and workshops; and (6) team performance feedback sessions.

**Figure 1.**
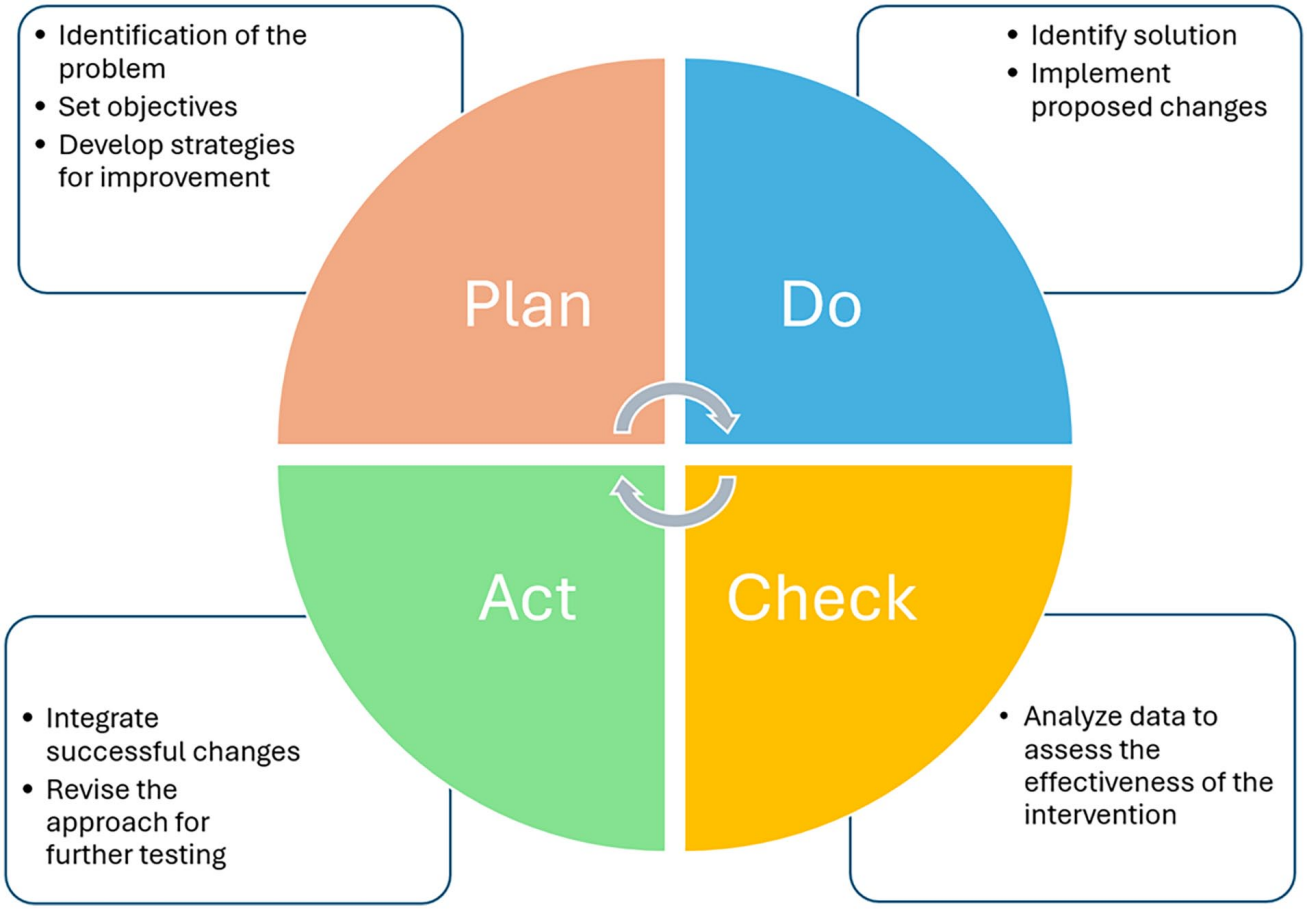
The Plan-Do-Check-Act cycle approach for quality and safety improvement adapted from Knudsen et al.^22^

According to the conceptual model of the Registered Nurses (RN) Association of Ontario^16^ and as advocated by the University Hospital Center (UHC), developing and sustaining transformational nursing leadership is imperative to address team dynamics, such as those of the acute geriatric care unit (AGCU) through a multidimensional approach to achieve quality and safety care objectives, and foster a stimulating environment that encourages collaboration.^3,11,17–21^ However, implementing a multidimensional approach, such as improving team dynamics, requires the adoption of a quality management framework to ensure its success and acceptability. The Deming Wheel model (Plan-Do-Check-Act (PDCA)) was adopted for this quality improvement project, first because of the recommendations of the National Hospital Association in using the PDCA framework for quality management, and second due to the iterative process of working in a team dynamic within the healthcare team.^22,23^ The PDCA cycle is a systematic, iterative method used to test and implement changes. The Plan phase involves identifying a problem, setting objectives, and developing a strategy for improvement. In the Do phase, the proposed changes are implemented. The Check phase involves analyzing data to assess the effectiveness of the intervention. Finally, the Act phase integrates successful changes into standard practice or revises the approach for further testing. This cyclical process fosters continuous quality improvement by enabling healthcare teams to refine interventions based on real-world feedback and data analysis (Figure 1).^22,23^

## Methods

This quality improvement project describes the team dynamic improvement process and its interventions implemented at AGCU using the PDCA framework, and to evaluate its impact on the healthcare team, patients, and organization, referring to the IPOI model.

The AGCU where this work was initiated, consists of 28 beds, divided into 3 sectors, dedicated to older polymorbid and polymedicated adults, requiring specialized care considering their age-related complexity. The unit is part of UHC that accommodates 1400 beds across several sites. The healthcare team consists of 19 RN and 17 healthcare assistants (HAs). The daily organization of nursing work within the unit is based on the collaboration of RN and HA. Between 2018 and 2020, the AGCU underwent significant changes impacting the team dynamic, as well as various quality and safety improvement projects. These changes included changes in its management team (nurse manager of service, head of medical service, and two clinical nurse specialists) and its healthcare team (annual turnover rate of 40%), as well as workflow reorganization during the COVID-19 pandemic.

**Figure 2.**
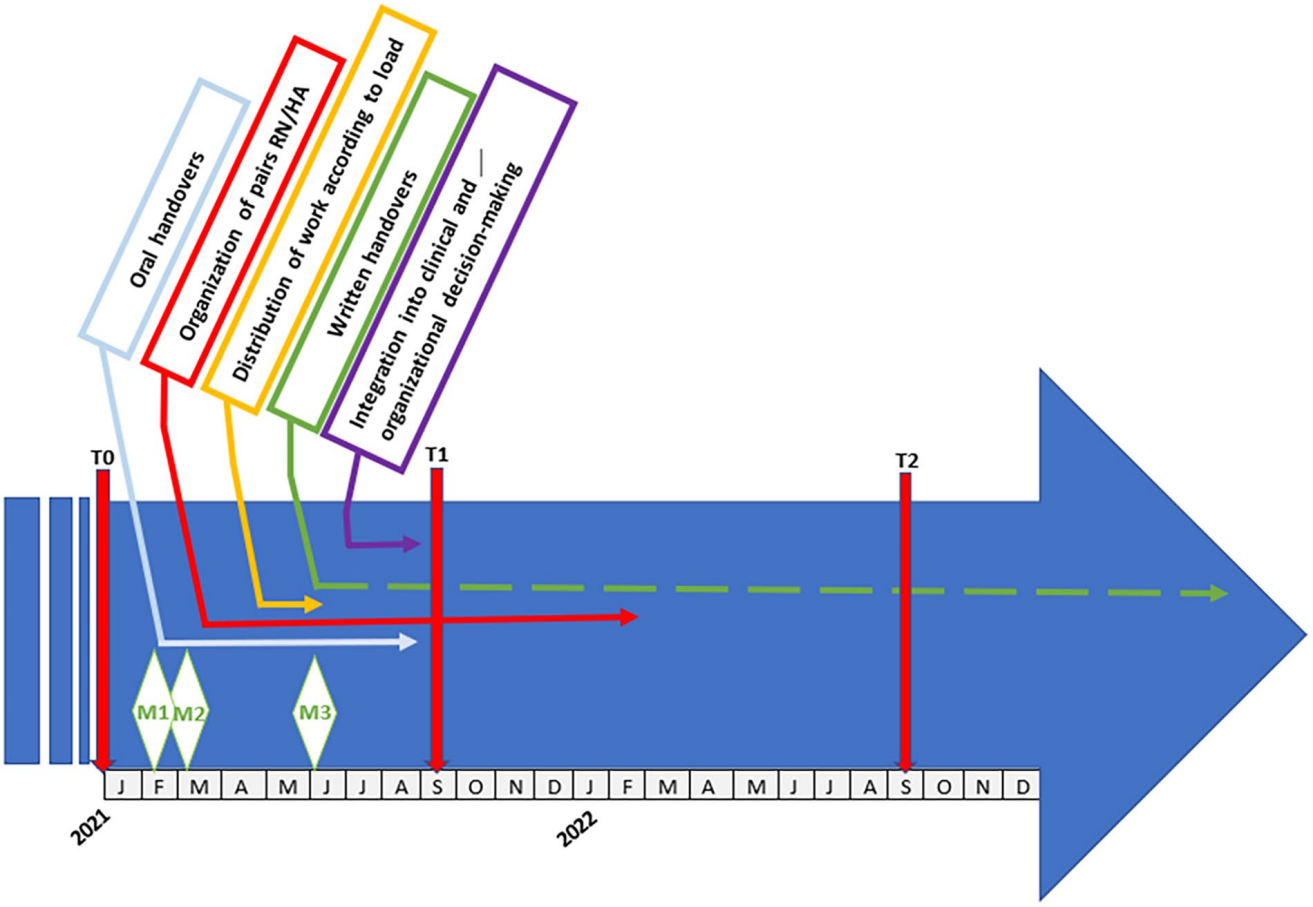
Planning and timeline of prioritized actions.

Figure 2 presents the main steps implemented during the process of work on team dynamics from the early part of 2021, namely: (1) data collection by questionnaires at T0, T1, and T2 submitted to the healthcare team, (2) team meetings M1, M2, and M3 dedicated to this process, and finally (3) the main actions for improvement as implemented (work on oral and written handovers, organization of the RN/HA pairs, etc.) with their durations represented by the vectors.

The structured process of team dynamics improvement followed the PDCA framework, ensuring continuous refinement and adaptation of interventions. In the Plan phase, an initial assessment (T0) was conducted in January 2021 using a self-administered and anonymous questionnaire to evaluate team dynamics and identify dissatisfaction areas (Appendix 1). This questionnaire is further described below. This step provided a baseline for the subsequent interventions. Following this, separate team meetings were organized: M1 in February 2021 with the nursing team and M2 in March 2021 with the HA team. These meetings allowed for the discussion of T0 results, the identification of dissatisfaction sources, and the formulation of preliminary solutions, setting the foundation for improvement actions. The Do phase involved the immediate implementation of identified interventions, such as clarifying roles and responsibilities, adjusting the distribution of care activities, and improving oral and written handovers through the implementation of standardized tools like TeamStepp^®^ (Figure 2). Midway through the implementation process, the Check phase was initiated with a second assessment (T2) to evaluate the effectiveness of the interventions and identify remaining challenges. The results of T2 were further analyzed during M3 in June 2021, where both RNs and HAs engaged in discussions to refine existing interventions and integrate healthcare team members into clinical and organizational decision-making. The process then advanced to the Act phase, where the final assessment (T3) measured the long-term impact of the interventions and ensured sustainability through structured monitoring mechanisms. During this phase, the final action plan was validated and refined in M3, incorporating cycles and subcycles of PDCA to support continuous improvement in team dynamics. The main interventions proposed by the IPOI model, described in the detailed action plan (Table 1), were addressed to achieve the improvement of team dynamics. The iterative nature of the PDCA framework ensured that each intervention was continuously assessed, adjusted, and optimized to enhance collaboration and efficiency within the healthcare team.

**Table 1.** Action plan and prioritization of interventions.

Issues	Intermediary issues	Objectives	Actions	Prioritization	Resources	Interventions according to IPOI model
Roles and responsibilities are not clearly defined/known. Lack of nursing leadership	Mutual lack of knowledge of RN and HA roles	Clarify the individual and common responsibilities and roles of each person (RN and HA)	Clarify individual and common responsibilities in the daily organization (specifications)	1	MT	Complex intervention and reorganization of the team
			Distribute upon arrival in the department the specifications of the two functions (reception/welcome file) during the integration week provide a moment for discussion during the integration week	2	CNS/UHN	Implementing tools
	Limited HA scope of practice	Working on and promoting patient-centered nursing leadership	Clarify the specific roles of the RNs and HAs	2	NM/UHN	Complex intervention
	Lack of nursing positioning/leadership		Prioritization of nursing care and monitoring by taking responsibility for all patients (work on nursing leadership)	2	CNS	Complex intervention and reorganization of the team
			Empowerment of care provided by HAs in collaboration with RNs	2	CNS/UHN	Reorganization of the team
Difficulty giving feedback to colleagues	Lack of communication in the pair RN-HA	Promote communication between RN-HA pairs	Strengthen RNs/HAs communication points during the working day for the organization of care through clarification of the organization	1	MT	Reorganization of meetings
			Inform the healthcare team of the objectives of the communication points	1	NM	Reorganization of meetings
			Monitor the evolution of the daily communication points. Presence of CNS/UHN/NM and provide randomly feedback on communication points	1	CNS/UHN/NM	Performance feedback/complex intervention
			Include during monthly meetings a point on team dynamics and communication within the RN-HA pair	1	NM	Reorganization of meetings /performance feedback
		Adjusting the RN-HA pair’s communication	Implementation of a weekly debriefing meeting (max 30 min) on the service organization RN-HA pair. Remind the objectives/give feedback if necessary. Time dedicated to express team’s difficulties/facilities	1	NM/UHN	Performance feedback/reorganization of meetings
Impossibility of documenting in EHR some care activities/difficulty ensuring continuity of care or observations due to partial documentation	Time-consuming and incomplete documentation	Optimize documentation in the EHR	Identify RNs and has to an EHR documentation referents	1	UHN/NM	Implementing tools
			Define the individual needs of each HA and RN for documentation in EHR through individual coaching	1	CNS/WG	Training
			Update the service documents references for service RN and HA concerning the EHR documentation	2	CNS/WG	Implementing tools
Work overload, lack of staff. Long time for interdisciplinary meetings, lack of time for complex patient situations	Current organization unsatisfactory	Optimize the general organization of the service	Reorganization of morning handover. Reorganization of care activities in two sectors at the same time rather 3 sectors	1	UHN/SRN	Reorganization of the team
			Determine transversal care activities	1	UHN/SRN/NM	
			Distribution of the care activities according to the day’s workload between sectors	1	UHN	
			Reorganization of scheduled medication preparation	2	CNS/SRN/WG	
			Evaluate the effective day’s workload	2	UHN/NM	
			Reorganization of medical-nurse ward rounds	3	MT	
			Reorganize the weekly interdisciplinary meeting	3	MT	Reorganization of meetings
		Strengthen the culture of mutual aid between RN-RN, HA-HA, and RN-HA and maintain the safety of care	Promote mutual assistance and encourage colleagues to offer/ask for help if necessary	1	UHN/SRN	Reorganization of the team
			Provide feedback during the daily morning. Communication points of RN-HA	1	UHN/SRN/CNS	Reorganization of meetings
			Team training in TeamStepp^®^ tools	1	CNS	Training
Lack of time to integrate new members of healthcare team	Lack of respect for the integration grid for new members of healthcare team	Strengthen the integration of new members of healthcare team at AGCU	Adjustment of the integration grid for new members of healthcare team	2	CNS	Implementing tools
			Routine planning of healthcare members’ coaching	2	CNS/UHN/NM	Training
			Organize a check-in on the integration of each new healthcare team members	2	CNS/UHN/NM	Training

RN: registered nurse; HA: healthcare assistant; MT: management team; CNS: clinical nurse specialist; UHN: unit head nurse; MN: nurse manager; SRN: substitute responsible nurse; EHR: electronic health record; WG: work group; 1 represents high priority and 3 represents lower priority.

Table 1 presents the action plan detailing all the interventions implemented, their prioritizations by the healthcare team, and in the last column, their concordance with the six types of interventions proposed by Körner et al.^15^ in the IPOI model. These interventions are summarized as follows:

- Complex interventions (e.g., work on patient-centered nursing leadership, clarification of individual and collective roles).
- Implementation of tools (e.g., improvement of oral handovers, implementing the TeamStepp^®^ oral handover tool, and improvement of written handovers by implementing a common documentation).
- Team reorganization (e.g., reorganization of RN-HA and distribution of care activities according to the day’s workload).
- Reorganization of team meetings (e.g., weekly debriefing, integration of team dynamic theme in monthly team meetings, interdisciplinary meetings, and RN-HA daily meetings).
- Training (e.g., coaching, integration of new collaborators).
- Performance feedback (e.g., weekly debriefing and reorganization of monthly meetings).

## Measures

This section addresses the data measured according to the IPOI model to assess the impact of the actions taken to improve team dynamics. The IPOI model presents 37 variables divided into 3 domains (Input (7 variables), Process (12 variables), and Output (18 variables)). The variables of the Output domain are categorized into three types: team (4 variables), patient (4 variables), and organization (10 variables). In this quality management project, pragmatically, we selected a limited number of output variables available in the institution. Table 2 presents the variables retained from the IPOI model and the existing institutional data.

**Table 2.** Variables retained according to the IPOI model and their sources.

Output	Sources
**Healthcare team**	
Satisfaction	Questionnaire
Performance	
• Pain management	Database^9^
• Assessment of pressure ulcer risk	Database^9^
• Absence	Database^9^
**Patient**	
Patient safety	
• Rate of pressure ulcers	Database^9^
• Pharmacological and non-pharmacological treatment of pain	Databse^9^
• Bladder catheter duration	Databse^9^
**Organization**	
Average length of stay	Service statistics
Time for team meetings	Unit statistics
Critical and adverse events reporting	Specific database for collecting adverse critical events (RECI)

In the absence of validated questionnaires, specifically in French, which assess the issues raised by the healthcare team during M, M2, and M3 meetings,^24^ a questionnaire was developed to measure the healthcare team’s degree of satisfaction with the team dynamic in the service and to explore the different issues of input and process dimensions of the IPOI model (Appendix 1). Initially, the questions were developed based on a comprehensive literature review on team dynamics, supplemented by informal feedback from the healthcare team to ensure relevance. The questionnaire was then independently reviewed by three experts involved in quality-of-care improvement projects within the department. Over three rounds, experts provided feedback, leading to iterative refinements to enhance clarity, relevance, and completeness. The responses to the questionnaire were formulated using a Likert scale with ratings from one (not at all satisfied) to ten (very satisfied).^25^ The questionnaire was validated through an expert review process, a widely recognized method for assessing content validity in the absence of psychometric testing.^26^ The new themes proposed by the healthcare team following the M1, M2, and M3 meetings were integrated into the T1 and T2 questionnaires. These included the healthcare team satisfaction within the RN-HA pair dynamic, the distribution of care activities according to the workload, and the integration of the healthcare team in clinical and organizational decision-making.

The questionnaire was self-administered anonymously in paper format and distributed to all healthcare team members as part of a convenience sample, with no exclusion criteria applied. All members were included in the study, comprising 19 RNs and 17 HAs. The questionnaires were placed in the lockers of each team member, and once completed, they were returned to a designated box at the nursing desk. Participation was entirely voluntary, with no obligation or pressure, and team members were informed during meetings about the study’s purpose. Consent was implied through the completion and submission of the questionnaire, those who chose not to participate simply did not respond. In addition, routine institutional indicator data measured within the service were extracted for the duration of this project. As all team members were included, no formal sample size calculation was performed.

### Institutional review board approval

This quality improvement project was approved by the Internal Medicine Department of the UHC. As the quality improvement project exclusively involved healthcare professionals, Institutional Review Board (IRB) approval and ethical review were not required.

### Statistical analysis

Data were analyzed using descriptive statistics, including numbers, averages, and percentages, with all statistical analyses performed in Excel^®^. The results are presented in the following section.

## Results

### Healthcare team characteristics

The participation rate varied between approximately 45% and 68%, with a very variable total experience (Table 3). The T0 results showed that most of the healthcare team (55%) had a professional experience of 2 years or less. This rate gradually decreased and 50% of the healthcare team had a total professional experience of 5 years or more in T2.

**Table 3.** Characteristics of the healthcare team.

Characteristics	January 2021 (T0)	September 2021 (T1)	September 2022 (T2)
Participation rate *n* (%)	20 (57.14%)	24 (68.57%)	16 (45.71%)
Total professional experience, *n* (%)			
0–2 years	11 (55%)	8 (33.33%)	3 (18.75%)
3–4 years	4 (20%)	7 (29.16%)	5 (31.25%)
5 years and more	5 (25%)	9 (37.5%)	8 (50%)
Geriatric professional experience, *n* (%)			
0–2 years	11 (55%)	8 (33.33%)	3 (18.75%)
3–4 years	4 (20%)	8 (33.33%)	5 (31.25%)
5 years and more	5 (25%)	8 (33.33%)	8 (50%)
Perception of the team of being sufficiently trained to work in geriatrics, avg. (SD)	7.65 (1.19)	7.58 (1.06)	8.40 (1.04)

Avg.: average; SD: standard deviation.

The following results are presented according to the IPOI model for (1) the healthcare team, (2) the patient, and (3) the organization.

### Healthcare team results

Globally, the healthcare team considered that they had a sufficient or even good level of training to work with older people (Table 3). The average scores for this question varied across the four questionnaires from 7.65 the lowest value in 2020 (T0) to 8.4 the highest value in 2022 (T2).

The results of the questionnaires demonstrated a constant improvement in the satisfaction of the healthcare team with oral handovers since January 2021 (Table 4). The average satisfaction scores increased by 1.35 points between January 2021 (T0) and September 2022 (T2).

**Table 4.** Healthcare team outcomes.

Outcomes	January 2021 (T0)	September 2021 (T1)	September 2022 (T2)
Oral handover satisfaction, avg. (SD)	5.90 (1,32)	7.21 (1.16)	7.25 (1.13)
Written handover satisfaction, avg. (SD)	4.85 (1.88)	4.83 (2.36)	4.63 (1.97)
Satisfaction with the dynamic of the RN-HA pair, avg. (SD)	NE	5.79 (2.09)	7.38 (1.50)
Satisfaction with the distribution of care activities according to workload, avg. (SD)	NE	5.79 (4.83)	7.38 (1.41)
Satisfaction with the integration of the healthcare team members in the clinical and organizational decision-making of the service, avg. (SD)	NE	5.75 (2.06)	6,50 (1.56)
Satisfaction with AGCU healthcare team dynamic, avg. (SD)	4.95 (2.36)	6.96 (1.63)	8.06 (1.45)
Assessment of pressure ulcer risk by Braden Scale within 24 h of admission, avg. (SD)	82.18 (5.55)	76.23 (5.25)	72.54 (7.68)
Pain assessment 4 h after admission, avg. (SD)	82.94 (4.89)	80.74 (5.28)	83.12 (5.40)
Absence rate excluding maternity, accidents, and legal obligations (%), avg. (SD)	6.30 (2.36)	3.30 (1.17)	3.94 (2.51)

Avg.: average; SD: standard deviation; NE: not evaluated.

The following results concern the new themes prioritized by the healthcare team during meetings M1, M2, and M3, which were not evaluated in January 2020 (T0).

A discernible enhancement was observed in the satisfaction of the healthcare team within RN-HA pair dynamics (Table 4), with an increase of 1.59 points in the average score from September 2021 (T1) to 1 year after the implementation of the action plan in September 2022 (T2).

The satisfaction of the healthcare team with the distribution of care activities according to workload, a theme that emerged and was prioritized in meetings M1, M2, and M3, demonstrated a clear improvement. The satisfaction average score increased by 1.59 from September 2021 (T1) to 1 year after in September 2022 (T2) (Table 4).

Considering the theme of the integration of the healthcare team in organizational and clinical decision-making, the results of the questionnaires indicated an improvement in the team’s satisfaction with their integration into decision-making following the implementation of actions considering this topic (Table 4). The average satisfaction scores increased by 0.75 points between September 2021 (T1) and September 2022 (T2).

Furthermore, the results showed a favorable impact on all the actions within the healthcare team dynamic, with an increased average score of 3.11 on this topic since January 2021 (T0) (Table 4) prior to the implementation of the action plan (T0), to September 2022 (T2), where the major actions were implemented.

On the other hand, the service’s routine data on absence rates, excluding maternity, accident, and legal obligations (e.g., military service), as well as training, demonstrated a reduction of approximately half of the annual average rate between 2020 and 2021, from 6.30% to 3.30%. This remained relatively stable in 2022 with 3.94% in 2022 (Table 4).

In addition, data relating to team performance, according to the IPOI model, indicated variable trends in the application of institutional clinical care processes for pain and pressure ulcer management (Table 4). Notably, the results showed an average decline of approximately 4% per year from 2020 to 2022 in the assessment of the risk of pressure ulcer rate using the Braden score within 24 h following admission. The average documentation of pain assessment within 4 h following patient admission, according to institutional clinical recommendations, demonstrated a slight improvement between 2020 and 2022 with an increase of 0.18% (Table 4).

### Patient outcomes

Routine data measuring patients’ outcomes extracted from the institutional dashboard concerning the annual average rate of nosocomial pressure ulcers demonstrated some stability between 2020 and 2022 (Table 5). The annual average rate of pressure ulcers of categories 2, 3, and 4 or more acquired in the service was stable and less than 2% from 2020 to 2022.

**Table 5.** Patients’ outcomes.

Outcomes	2020	2021	2022
Rate of pressure ulcers acquired in the unit category 2, 3, and 4 and more (%), avg. (SD)	1.30 (1.08)	1.29 (0.86)	1.83 (1.00)
Rate of pharmacological and non-pharmacological interventions during the pain period (%), avg. (SD)	94.03 (3.18)	88.33 (5.03)	85.69 (7.69)
Duration in days of maintaining a bladder catheter, avg. (SD)	5.90 (3.07)	5.36 (2.49)	4.75 (0.79)

Avg.: average; SD: standard deviation.

Considering pharmacological and non-pharmacological pain management for hospitalized patients, data extracted from the institutional database indicated that the interventions deployed during pain periods decreased at an average rate of 8.34% from 2020 to 2022 (Table 5).

Furthermore, data regarding the duration of maintaining bladder catheters during the hospital stay showed an improvement prior to the deployment of this team dynamic work in 2020–2022, with a decrease of almost 1 day (Table 5).

### Organizational outcomes

The organizational outcomes concern the length of acute (A) and rehabilitation (B) stays, the number of meetings with the healthcare team, and the number of adverse events reported by members of the healthcare team.

The average length of acute and rehabilitation stays (A and B) showed a discernible trend downwards with a reduction of approximately 1.55 days, respectively, between 2020 and 2021 and remained stable at 5.70 days in 2022 (Table 6).

**Table 6.** Organization’s outcomes.

Outcomes	2020	2021	2022
Average length of stays A and B, avg. (SD)	6.75 (2.15)	5.70 (1.2)	5.70 (1.1)
Number of meetings with the healthcare team, avg. (SD)	1.67 (0.56)	2.75 (1.17)	2.83 (0.89)
Number of adverse event reports, avg. (SD)	1.25 (1.04)	1.50 (0.67)	1.67 (1.00)

Avg.: average; SD: standard deviation.

Furthermore, the average number of meetings with the AGCU healthcare team also increased since 2020, rising from an average of 1.67 to 2.75 in 2022 (Table 6).

In addition, routine institutional data from the electronic health record (EHR) dashboard of the average number of adverse events reported by the AGCU healthcare team has increased by approximately 0.5 from 2020 and 2022 (Table 6).

## Discussion

This article aims to demonstrate the impact of improving healthcare team dynamics on the healthcare team, patients, and organizational outcomes.

Healthcare team satisfaction with oral handovers showed a constant improvement since the reorganization of oral handovers initiated after the T0 audit and the M1 meeting. These results are consistent with those of Nasiri et al.,^18^ which demonstrated an increase in satisfaction with oral handovers when restructured. In this work, both oral and written handovers were structured, standardized, and simplified. However, the satisfaction of written handovers in this project demonstrated only a relative improvement despite the actions implemented since May 2021 (following the two meetings M1 and M2). This may be due to the limited changes possible regarding the harmonization of documentation in the EHR. This process includes the involvement of the information systems department with limited resources. While EHR is designed to provide ergonomic documentation to healthcare teams in ensuring the safe clinical monitoring of patients, requests from healthcare teams for EHR developments are rarely considered. This failure to meet healthcare team expectations in this regard remains a source of their dissatisfaction. On the other hand, it is also important to note that the work of harmonizing the documentation in EHR is still in progress and not yet finalized.

Furthermore, the organization of daily healthcare activities in the AGCU has been reorganized according to the workload, rather than by sectors as it was usually done. This potentially promoted fairness and mutual assistance between healthcare team members and reduced work exhaustion to a certain extent. The results indicated a positive impact of this new reorganization on the healthcare team regarding the distribution of healthcare activities according to workload. This finding aligns with the conclusions of both McClain et al.^11^ and Rosen et al.^12^ increasing a healthy, collaborative, equitable, stimulating, and flexible work environment to increase healthcare team engagement.

The implementation of the monthly meetings with the AGCU management team and the interprofessional meetings to address clinical and management questions raised by the healthcare team enabled an increase in the satisfaction of healthcare members regarding their integration in clinical and organizational decision-making. This contributes to the involvement of healthcare members and their commitment to AGCU service as promoted by the transformational leadership model.^16^ Furthermore, the AGCU’s routine data measurement of absences, excluding maternity, accident, and legal obligations, indicated notable improvement in the average absence days following the implementation of the interventions to improve team dynamics. This reflects the work of Schreuder et al.^13^ on the relationship between short-term absences and the type of leadership and it is in line with the findings of D’Amour et al.^10^ and Wei et al.,^14^ who highlighted the importance of the collaborative context. The results of this work demonstrated that all the initiatives of the action plan to enhance the context of collaboration, such as the integration of the healthcare team in decision-making and the reorganization of care activities according to the workload, have contributed to an improvement in the level of healthcare team satisfaction regarding the team dynamic. These results also correspond to those of Nantsupawat et al.^1^ and Song et al.^2^ in their studies.

Regarding the institutional clinical care processes for pressure ulcer management, the routine data concerning pressure ulcer management, which encompass the risk assessment and the implementation of pressure ulcer prevention interventions in a high-risk population, were globally satisfactory. Although the risk assessment in the 24 h following admission has decreased annually since 2020, the average annual rate of pressure ulcers in categories 2, 3, and 4 and above remained relatively stable and lower than what is described in the literature for the same population.^27^ This may also be attributed to the multicomponent approach of this theme, specifically dedicated to improving the management of pressure ulcers in the AGCU since 2015. However, the moderate decrease in the documented pressure ulcer risk assessment rate could be explained by its direct relationship to the satisfaction with the documentation in her, given that the Braden score has to be completed using a form only available in the EHR.

The other clinical process monitored was pain management. The routine data showed a certain stability in the assessment rate within 4 h of patients’ admission, and these rates are comparable to those of Minaya-Freire et al.^17^ in an acute geriatric unit. However, it is challenging to make a comparison concerning the pharmacological and non-pharmacological interventions deployed in the AGCU during pain episodes, as Minaya-Freire et al.^17^ did not specify this aspect in their study. Nevertheless, according to the results of this study, the rates of pharmacological and non-pharmacological interventions demonstrated high and relatively stable rates. As with pressure ulcers, pain management in AGCU has received particular attention since 2019, which could contribute to the satisfactory results of both the initial pain assessment within the 4 h period and the pharmacological and non-pharmacological management during pain episodes. Furthermore, the duration of maintenance of bladder catheters has decreased since 2020. Namely, a project specifically for the management of functional urinary disorders had started in the department in 2015. This work in the improvement of team dynamics reinforced the application of best practices by the healthcare team.

According to the IPOI model of Körner et al.,^15^ the average length of hospital stay is an organizational indicator (Output). Although the length of stay has decreased since 2020, this cannot be exclusively attributed to the work on team dynamics carried out at AGCU. Despite several interventions of the action plan aimed to improve nursing processes and therefore the efficiency of care, the concomitance with other medical and organizational initiatives in the service to improve length of stay makes it challenging to attribute these results only to the action plan on the team dynamic.

On the other hand, the increase in meetings with the healthcare team must be contextualized. During the period of 2020 and 2021, which was marked by the COVID-19 pandemic, group meetings were strongly discouraged or even prohibited.

In addition, the reporting of adverse events depends on the diligence of its documentation in the EHR and on the number of adverse events occurring, which can vary from one period to another. The relative increase observed may be explained by the interventions initiated in the service. This reflects the climate of trust established following the improvement of team dynamic interventions initiated in the AGCU, which were favorable to the reporting of adverse events by the healthcare team without fear of consequence.

Usually, improving the quality of care focuses only on the improvement of care processes. This project allowed us to adopt a more comprehensive approach, applying a theoretical model that enables multimodal fields of action, thus promoting a collaborative and stimulating work environment for the healthcare team to impact patient, healthcare team, and organizational outcomes.

This work also permitted the observation of the team’s involvement in the development of concrete actions to enhance their work environment and ensure that patients receive quality and safety care. Furthermore, this work has also initiated a proactive multidisciplinary approach in the service, such as working on the transition of patients hospitalized in the AGCU.

### Strengths and limitations

This work has some strengths and limitations. One of the strengths of this work consists of the collaborative process implemented according to the transformational leadership model promulgated by the Registered Nurses Association of Ontario.^16^ Moreover, the use of the iterative PDCA model throughout the process gave an evolving and adaptable approach to the needs and priorities of the healthcare team. Another strength of this work is the comprehensive approach to the process of improving team dynamics. The results were not limited only to measuring the healthcare team satisfaction but also highlighted its impact on the patient, healthcare team, and organizational outcomes extracted from the routine data available according to the IPOI model of Körner et al.,^15^ which contributes to promote nurse-sensitive indicators in healthcare systems.^7–9^

Some limitations of this quality-of-care improvement project should be noted. The first limitation consists of the limited availability of routine institutional outcomes to cover all the outcomes proposed by the IPOI model of Körner et al.^15^ The second limitation was the difficulty to find elements of comparison for some themes, such as satisfaction with the dynamics of the RN-HA pair, the distribution of care activities according to the workload, and the integration of the healthcare team in clinical and management decision-making. These themes emerged after the M1, M2, and M3 meetings.

Another limitation of this study was the small sample size (19 RNs and 17 HAs), which affected the generalizability of the findings. Since all available healthcare team members were included, no formal sample size calculation was conducted, which may impact the interpretation and applicability of the findings. In addition, participation dropped by nearly 11% at T2, mainly due to fluctuations in staff presence during data collection rather than a lack of interest. While this reduction may have influenced the results, the overall trends remain representative of the AGCU team, as confirmed by discussions with team members during the presentation of study findings. In addition, the post-COVID-19 period may have impacted some results, such as absenteeism. Lastly, a limitation of this study is that the questionnaire used was not formally validated through psychometric testing. Although it was reviewed and adapted by experts in the field, it was not pilot-tested. This lack of formal validation could affect the reliability and generalizability of the results.

Based on the results of this work, some perspectives and implications for practice could be considered in the quality and safety of care improvement process. The improvement of the quality and safety of care approach should integrate the component of improving team dynamics. This should be working on care processes and the creation of a collaborative and stimulating work environment for healthcare team members at the same time. Appropriate models could be developed, tested, and used in an integrative approach to evaluate their impact not only on care teams but also on patients and organizations. Evaluation of their impacts on the healthcare teams, patients, and organizations would maintain the quality of care while preserving the care teams.

## Conclusion

The implementation of actions aimed at improving team dynamics increased the overall quality of outcomes. Healthcare team satisfaction increased and contributed to the reduction in absences excluding maternity, accidents, and legal obligations. The organization’s outcomes have also seen improvements regarding length of stay, team meetings, and reporting of adverse events by the healthcare team. The patients’ outcomes remained highly satisfactory and relatively stable throughout the process, notably in terms of pressure ulcers and pain management in the service. Consequently, we argue that the integration of improving healthcare team dynamics in the processes of improving the quality and safety of care has to be considered to optimize clinical and organizational outcomes.

## Supplemental Material

sj-docx-1-smo-10.1177_20503121251333314 – Supplemental material for Improving team dynamics in an acute older people care unit to improve quality and safety of care Supplemental material, sj-docx-1-smo-10.1177_20503121251333314 for Improving team dynamics in an acute older people care unit to improve quality and safety of care by Rachid Akrour, Anja Pattschull, Suzana Stamenkovic, Catherine Courret Gilgen, Alberto Garcia Manjon, Yvan Bourgeois and Joachim Rapin in SAGE Open Medicine
