# Supplementary material for: Improving team dynamics in an acute older people care unit to improve quality and safety of care
Source: SAGE Open Med. 2025 Apr 27;13:20503121251333314. doi: 10.1177/20503121251333314 (PMC12035220; doi:10.1177/20503121251333314)
Supplement: sj-docx-1-smo-10.1177_20503121251333314 – Supplemental material for Improving team dynamics in an acute older people care unit to improve quality and safety of care [file sj-docx-1-smo-10.1177_20503121251333314.docx]

| **Function** |  |  | | HA |  |  | RN |  |  | |  |  |  |  |  |  |  |  |  |
| --- | --- | --- | --- | --- | --- | --- | --- | --- | --- | --- | --- | --- | --- | --- | --- | --- | --- | --- | --- |
|  |  |  | |  |  |  |  |  |  | |  |  |  |  |  |  |  |  |  |
| **Total Professional experience** | | | | 0-2 y |  |  | 3-4 y |  | 5 y and more | | | | |  |  |  |  |  |  |
|  |  | |  |  |  |  |  |  |  | |  |  |  |  |  |  |  |  |  |
| **Geriatric professional experience** | | | | 0-2 y |  |  | 3-4 y |  | 5 y and more | | | | |  |  |  |  |  |  |
|  |  |  | |  |  |  |  |  |  | |  |  |  |  |  |  |  |  |  |
| ***Circle the answer that suits you*** | | | | | | | | | | | | | | | | | | | |
| **Scope of practice and skills** | | | | | | | | | | | | | | | | | | | |
|  |  |  | |  |  |  |  |  |  | |  |  |  |  |  |  |  |  |  |
|  |  |  | |  |  |  |  | **Not agree at all** | | |  |  |  |  |  |  |  |  | **Totally agree** |
| **I feel sufficiently trained to work and care for geriatric patients** | | | | | | | | | **1** | | **2** | **3** | **4** | **5** | **6** | **7** | **8** | **9** | **10** |
|  |  |  | |  |  |  |  |  |  | |  |  |  |  |  |  |  |  |  |
| *If you encounter any obstacles/difficulties, please cite them* | | | | | | | | | | | | | | | | | | | |
|  |  |  | |  |  |  |  |  |  | |  |  |  |  |  |  |  |  |  |
| **Written and oral handovers** | | | | | | | | | | | | | | | | | | | |
|  |  |  | |  |  |  |  |  | **Not at all satisfied** | |  |  |  |  |  |  |  |  | **Totally satisfied** |
| **Are you satisfied with the documentation in the EHR?** | | | | |  |  |  |  | **1** | | **2** | **3** | **4** | **5** | **6** | **7** | **8** | **9** | **10** |
|  |  |  | |  |  |  |  |  |  | |  |  |  |  |  |  |  |  |  |
| **Are you satisfied with the oral handover?** | | | | | |  |  |  | **1** | | **2** | **3** | **4** | **5** | **6** | **7** | **8** | **9** | **10** |
| *Comments/suggestions* | | | | | | | | | | | | | | | | | | | |
|  |  |  | |  |  |  |  |  |  | |  |  |  |  |  |  |  |  |  |
| **Interprofessionality** | | | | | | | | | | | | | | | | | | | |
|  |  |  | |  |  |  |  |  | **Not at all satisfied** | |  |  |  |  |  |  |  |  | **Totally satisfied** |
| **Are you satisfied with the medical-nursing ward rounds?** | | | | | | | | | **1** | | **2** | **3** | **4** | **5** | **6** | **7** | **8** | **9** | **10** |
|  |  |  | |  |  |  |  |  |  | |  |  |  |  |  |  |  |  |  |
| **Are you satisfied with the weekly inter-professional meetings?** | | | | | | | | | **1** | | **2** | **3** | **4** | **5** | **6** | **7** | **8** | **9** | **10** |
|  |  |  | |  |  |  |  |  |  | |  |  |  |  |  |  |  |  |  |
| **Are you satisfied with the interprofessional collaboration?** | | | | | | |  |  | **1** | | **2** | **3** | **4** | **5** | **6** | **7** | **8** | **9** | **10** |
|  |  |  | |  |  |  |  |  |  | |  |  |  |  |  |  |  |  |  |
| *Comments/suggestions* | | | | | | | | | | | | | | | | | | | |
|  |  |  | |  |  |  |  |  |  | |  |  |  |  |  |  |  |  |  |
| **Healthcare team - RN-HA pairs** | | | | | | | | | | | | | | | | | | | |
|  |  |  | |  |  |  |  |  | **Not included at all** | |  |  |  |  |  |  |  |  | **Totally included** |
| **Do you feel included in clinical and organizational decision-making at AGCU?** | | | | | | | | | **1** | | **2** | **3** | **4** | **5** | **6** | **7** | **8** | **9** | **10** |
|  |  |  | |  |  |  |  |  |  | |  |  |  |  |  |  |  |  |  |
|  |  |  | |  |  |  |  |  | **Not at all satisfied** | |  |  |  |  |  |  |  |  | **Totally satisfied** |
| **Are you satisfied with the team dynamic at AGCU?** | | | | |  |  |  |  | **1** | | **2** | **3** | **4** | **5** | **6** | **7** | **8** | **9** | **10** |
|  |  |  | |  |  |  |  |  |  | |  |  |  |  |  |  |  |  |  |
| **Are you satisfied with the distribution of care activities according to workload?** | | | | | | | | | | **1** | **2** | **3** | **4** | **5** | **6** | **7** | **8** | **9** | **10** |
|  |  |  | |  |  |  |  |  |  | |  |  |  |  |  |  |  |  |  |
| **Are you satisfied with the organization of the pair (RN/HA)?** | | | | | | | | | **1** | | **2** | **3** | **4** | **5** | **6** | **7** | **8** | **9** | **10** |
|  |  |  | |  |  |  |  |  |  | |  |  |  |  |  |  |  |  |  |
| **Are you satisfied with the internal communication of the RN-HA pair?** | | | | | | | |  | **1** | | **2** | **3** | **4** | **5** | **6** | **7** | **8** | **9** | **10** |
| *Comments/suggestions* | |  | |  |  |  |  |  |  | |  |  |  |  |  |  |  |  |  |
